# Self-rated health in late adolescence as a predictor for mortality between 46 and 70 years of age

**DOI:** 10.1038/s41598-024-75158-x

**Published:** 2024-10-15

**Authors:** Anna Andreasson, Emelie Thern, Tomas Hemmingsson

**Affiliations:** 1 Department of Psychology, Stockholm University, Stockholm, Sweden; 2 Department of Clinical Neuroscience, Karolinska Institutet, Stockholm, Sweden; 3 School of Psychology, Macquarie University, North Ryde, NSW Australia; 4 Department of Public Health, Stockholm University, Stockholm, Sweden; 5 Institute of Environmental Medicine, Karolinska Institutet, Stockholm, Sweden; 6 Division of Psychobiology and Epidemiology, Department of Psychology, Stockholm University, Stockholm, Sweden

**Keywords:** Self-rated health, Mortality, Psychological factors, Health behaviors, Health status, Subjective health, Self-assessed health, Epidemiology, Outcomes research

## Abstract

Self-rated health is a common assessment used in epidemiological research and an independent predictor of morbidity and mortality. We investigate if a single measure of self-rated health in late adolescence predict mortality between 46 and 70 years of age. This study was based on 47 286 Swedish men that conscribed 1969–1970 at age 18–20 and that were still alive in 1997. Self-rated health and data on potential explanatory factors (psychological factors, health status markers and health behaviors) were collected at conscription. Adult socioeconomic position in 1990 was derived from registries. Death and cause of death (cancer, cardiovascular disease, violent and alcohol abuse related disorders) were derived from the Causes of Death Register between 1997 and 2019. Conscripts that rated their health as fair or poor/very poor had significantly higher hazard of all-cause mortality than conscript that rated their health as very good (HR_fair_ = 1.27, 95%CI:1.18–1.37 and HR_poor_ = 1.25, 95%CI:1.11–1.41) and disease-specific mortality. Adjusting for all explanatory factors attenuated the risk estimates by 9–100%. In conclusion, poor self-rated health reported in late adolescence predicts all-cause and disease-specific mortality between the ages of 46 and 70 years. Psychological factors and health behaviors measured at conscription may serve as potential explanatory factors underlying the predictive ability of self-rated health in late adolescence.

**Supplementary Information:**

The online version contains supplementary material available at 10.1038/s41598-024-75158-x.

## Introduction

Self-rated health is a common assessment in epidemiological research and poor self-rated health has been found to be an independent predictor of morbidity and mortality. For example, poor self-rated health was found to be the strongest mortality predictor of all in men and the third strongest in women in UK biobank^1^. The predictive ability is found across the world, in different ethnic regions, in both women and men, and across different age groups and is often independent of objective health measures and disease markers^2–4^.

The background of the independent predictive abilities of self-rated health is still poorly understood. Mechanisms may include in depth knowledge of personal and family medical history that is otherwise difficult to capture, knowledge of health behaviors, and symptoms that may be related to underlying disease processes^5^. Poor self-rated health has previously been associated with circulating levels of inflammatory makers^6,7^, supporting the theory that self-rated health may capture underlying disease processes. In a longitudinal study it was found that, although there was an overall association between proinflammatory cytokine interleukin-6 (IL-6) and self-rated health (i.e. in-between person), change in IL-6 was not correlated with changes in self-rated health within person suggesting that inflammatory state and poor self-rated health to some extent is a trait^8^. This is corroborated with genetic studies associating self-rated health with single nucleotide polymorphisms associated with immune function^9^, suggesting that self-rated health in part is an inherited trait and the predictive ability of self-rated health may be relatively stable over time. Lorem and coworkers studied self-rated health as a predictor of mortality compared with other health measures and observation time^3^. They found that self-rated health predicted mortality independent of health measures and that, although self-rated health is a subjective and time-dependent instrument and the predictive ability declined with time, self-rated health is still a stable measure over time^3^.

We have previously shown that self-rated health rated by men in their late adolescence predict mortality after 27 years of follow-up^10^. During this follow-up period, a large proportion of the deaths (70%) was classified as violent (i.e., due to suicide and accidents). Self-rated health showed the strongest prediction of alcohol and drug-related mortality and three psychological factors (low emotional control evaluated by psychologist, psychiatric diagnosis at conscription, and self-reported medication for nervous problems) were found to account for a significant part of the predictive ability of self-rated health. In our previous study, the predictive ability of self-rated health remained stable during the 27 year follow-up^10^. Less is however known about the predictive ability of self-rated health among adolescent men later in life when age related and natural causes of death are more common than violent deaths. In addition, it is not known if explanatory factors for the predictive abilities may change as the causes of death change with increasing age.

The aim of the present study was to investigate if an additional 23 years, altogether 50 years, of follow-up, alters the predictive ability of self-rated health, in strengths and in explanatory factors, as the causes of death changes as the men have aged. We hypothesized that self-rated health would still be a mortality predictor, but that psychological factors will explain less of the predictive capacity as the cause of death changes from a large proportion violent deaths to a large proportion of more natural causes of death such as cancer and cardiovascular disease.

## Methods

### Participants

We used data from a nation-wide, population-based study conducted during 1969–1970 of all Swedish men enlisted for conscription. During that time, conscription was mandatory in Sweden, and only 2–3% of men were exempted from conscription, mostly due to severe disabilities or diseases. This study was based on 49 132 Swedish men, age 18–20, included at that time.

All conscripts underwent an extensive health examination with height and weight measurements, personal interviews, and filled out questionnaires on self-rated health and health behaviours, at the time of conscription.

In the current study, men that died before 1997 (n = 1545 men), had missing information on self-rated health (n = 301) were excluded. The final analytical sample consisted of 47 286 men.

This study was approved by the Swedish Ethics Authority (approval No 2019–02161) and all methods were carried out in accordance with relevant guidelines and regulations. Informed consent was exempted by the Swedish Ethical Review Authority due to the character of the database and the anonymization of all data.

**Fig. 1:**
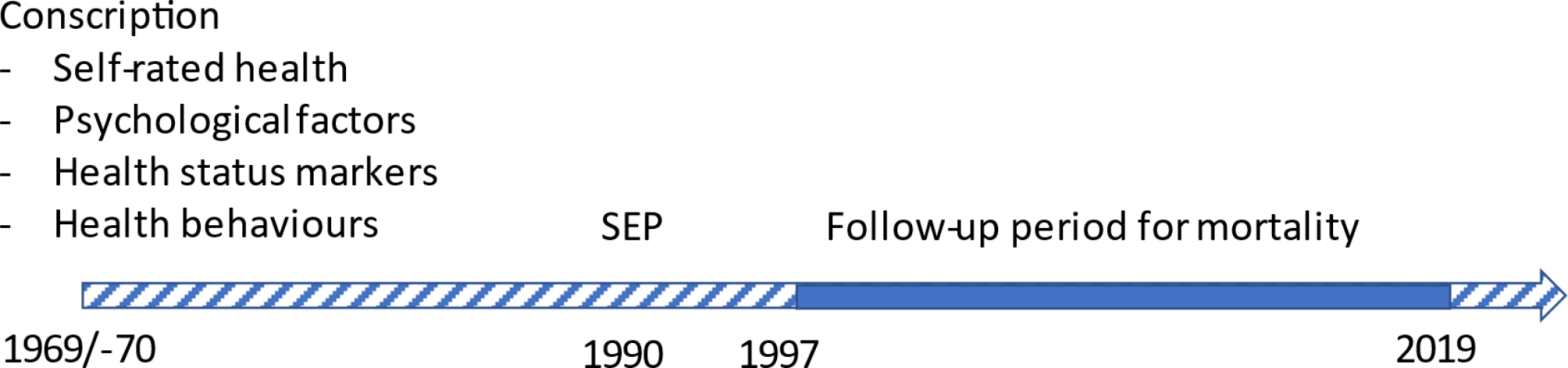
Overview of study.

An overview of the study is given in Fig. 1.

### Variables

#### Explanatory variable: self-rated health

Self-rated health was assessed at the time of conscription using the question “In general, would you say your health right now is: Very good (1), Good (2), Fair (3), Poor (4) or Very poor (5)?” Categories 4 and 5 were combined in the Cox regression analysis as few conscripts rated their health as poor.

#### Outcome variables: mortality

Information on mortality was obtained between 1997 to 2019 from The Causes of Death Register (CDR). The CDR contains data from 1961 on the cause of death for all Swedish citizens, including if the person died abroad. It is mandatory for the responsible physician to report the cause of death as well as contributing causes. Data on mortality was linked to the conscription cohort from CRD using the Swedish personal identification number. In addition to all-cause mortality, mortality per cause of death was investigated and was divided into natural causes i.e., cancer (total cancer, colon cancer, prostate cancer, lung cancer and stomach cancer) and cardiovascular disease (CVD) (ischemic heart disease (IHD) and stroke), violent causes (suicide and accidents) and alcohol abuse-related causes. Diagnostic codes are listed in supplement Table 1. An individual may have several causes of death why there are more specific causes of death than cases of all-cause mortality.

**Table 1:** Baseline characteristics of study population, stratified by self-rated health.

		Self-rated health				
	Total *n*(%)	Very good *n*(%)	Good *n*(%)	Fair *n*(%)	Poor or very poor *n*(%)	*p*-value
Total	42 286	19 272 (40.8)	19 619 (41.5)	6558 (13.9)	1834 (3.9)	
Socioeconomic position 1990 Unskilled worker Skilled worker Low-level non-manual employee Intermediate non-manual employee High-level non-manual employee Farmer Not classified	8989 (19.0) 9847 (20.8) 5007 (10.6) 8723 (18.5) 7675 (16.2) 1062 (2.3) 5983 (12.7)	3548 (18.4) 3821 (19.8) 2095 (19.9) 3768 (19.6) 3279 (17.0) 432 (2.2) 2332 (12.1)	3725 (19.0) 4312 (22.0) 2075 (10.6) 3609 (18.4) 3110 (15.9) 470 (2.4) 2308 (11.8)	1352 (20.6) 1404 (21.4) 654 (10.0) 1078 (16.4) 968 (14.8) 139 (2.1) 963 (14.7)	354 (19.3) 310 (16.9) 183 (10.0) 268 (14.6) 318 (17.3) 21 (1.2) 380 (20.7)	< 0.001
*Psychological factors*						
Low emotional control	14 076 (29.8)	3238 (16.8)	6169 (31.4)	3418 (52.2)	1251 (68.2)	< 0.001
Psychiatric diagnosis	5482 (11.6)	1026 (5.3)	2137 (10.9)	1582 (24.1)	737 (40.2)	< 0.001
Nerve medication	5279 (11.2)	1090 (5.7)	2173 (11.1)	1330 (20.5)	686 (37.8)	< 0.001
*Health status markers*						
High blood pressure	1470 (3.1)	565 (2.9)	650 (3.3)	195 (3.0)	60 (3.3)	
Erythrocyte sedimentation rate, mean(SD)	3.1 (3.7)	2.8 (3.3)	3.1 (3.6)	3.3 (4.1)	4.1 (6.0)	< 0.001
BMI 25-29.9 ≥30	2708 (5.7) 369 (0.8)	1049 (5.4) 122 (0.6)	1143 (5.8) 162 (0.8)	406 (6.2) 73 (1.1)	110 (6.00) 12 (0.7)	0.004
*Health Behaviors*						
Risky alcohol use	9925 (21.0)	3381 (17.5)	4151 (21.2)	1789 (27.3)	604 (32.9)	< 0.001
Smoking 1–5 cigarettes/day More than 5 cigarettes/day	5255 (11.1) 21 913 (46.3)	2321 (12.0) 7812 (40.5)	2175 (11.1) 9421 (48.0)	593 (9.0) 3598 (54.9)	166 (9.1) 1082 (59.0)	< 0.001
Cardiovascular fitness, mean(SD)	5.08 (2.8)	5.4 (2.9)	5.0 (2.7)	4.5 (2.6)	4.2 (2.7)	< 0.001

#### Explanatory factors

Data on explanatory factors that may explain the association between self-rated health and mortality were collected at the time of conscription. These factors were selected a priori and divided into three groups: psychological factors, health behaviors and health markers.

*Psychological factors* included low emotional control and psychiatric diagnoses as reported by the psychologist during the military service suitability assessment, and self-reported medication for nervous problems. Low emotional control was defined as having anxiety and/or low stress tolerance, having problems controlling nervousness, anxiousness, or aggression, and reduced functioning due to psychosomatic symptoms. Psychiatric diagnoses were defined according to the International Classification of Diseases version 8 (ICD-8); codes 290–315. Information of medication for nervous problems was obtained from the question “Have you been on medication for nervous problems? (1) Yes, several times, (2) Yes, sometimes, (3) No, never”, where several times and sometimes were combined into one category for the analyses. These covariates were included as these were the most prominent explanatory factors in the previous study on mortality prediction with 29 years follow-up, accounting for 84% of the association^10^.

*Health status markers* included BMI, erythrocyte sedimentation rate (ESR) and blood pressure. BMI was calculated (kg/m^2^) from height and weight taken at the conscription visit. Men with a BMI between 25 and 29. 9 were considered as overweight and men with a BMI over 30 were considered obese. ESR is a generic marker of an immune response and higher levels have been associated with poorer self-rated health in this study^7^. In total, 9% of the conscripts had any inflammatory diagnosis including hay fever, asthma, infection and inflammation in skin, infectious disease, diabetes mellitus, gastrointestinal inflammation, malignancies, lymphatic and haematopoietic tumours, arthritis and rheumatoid arthritis^7^. ESR was measured according to standard laboratory procedures (Westergren method) at the time of conscription and the ESR values were corrected for the hematocrit according to the formula ESR*Htc/45. Blood pressure was taken at the medical assessment during conscription, where a systolic/diastolic blood pressure 140/90 mmHg or higher was used as the cut off for high blood pressure.

*Health behaviors* included alcohol risk use, smoking and cardiovascular fitness. Alcohol risk use was defined as consuming at least 250 g of 100% alcohol per week or responding confirmative of any of four items in the questionnaire included being intoxicated often/quite often, being hungover often, sometimes drinking to alleviate hangovers or being apprehended for being drunk^11^. Smoking at the time of conscription was classified as either non-smoker, light smoker (smoking less than 5 cigarettes per day) or smoker (smoking at least five cigarettes per day). Cardiovascular fitness was tested on an ergometer cycle and maximum work capacity was divided by body weight, and transformed into a numeric scale 1 to 9, a higher number indicate a better cardiovascular fitness^12^.

Data on *socioeconomic position in 1990* when the conscripts were approximately 40 years old was obtained from the Swedish National Population and Housing censuses. The socioeconomic positions are Unskilled worker, Skilled worker, Low-level non-manual employee, Intermediate non-manual employee, High-level non-manual employee, Farmer and unclassified. Socioeconomic position was included as poorer self-rated health in adolescence may lead to a less favorable career that in turn may explain the association with mortality.

### Statistics

Cox regression analyses were performed to obtain hazard ratios (HR) with 95% confidence intervals (CI) using self-rated health as explanatory variable (four levels, very good as reference) and mortality as the outcome variable for the period 1997–2019. Firstly, crude models were calculated with all-cause, cancer, CVD, violent and alcohol abuse related deaths. Secondly, to identify factors that may serve as potential mechanisms underlying the predictive ability of self-rated health, socioeconomic position and psychological factors, health status markers and health behaviors, respectively, and in total, were added to the models as covariates.

The contribution of explanatory factors in explaining the self-rated health and mortality was determined by the percent attenuation in the hazard ratio for self-rated health after the inclusion of the risk factor in question. The percentage of HR reduction was calculated as ((HRcrude- HRadjusted)/ (HRcrude-1)) *100.

Thirdly, the follow-up period was divided into two to see if the predictive ability changed during the last two decades (1997–2008 and 2009–2019) of follow-up.

Missing values on covariates were coded as separate categories. All analyses were computed using Stata Statistical Software: Release 17.

## Results

Study group characteristics and covariates at the time of conscription are presented in Table 1. At conscription 3.9% (1834 individuals) reported their health as poor/very poor (combined), 13.9% (6558 individuals) as fair, 41.5% (19,619 individuals) as good and 40.8% (19,272 individuals) as very good.

Of the 49 132 conscripts, 7548 men died during the 50 years follow up: 1545 men died during the first 27 years of follow up before 1997, and 6003 died after 1997 and until end of follow-up in 2019. The most common cause of death during the later follow-up period was cancer (n = 2238) and CVD (n = 2643), Table 2.

**Table 2:** Distribution of outcomes 1997–2019, stratified by self-rated health. The diagnostic codes per outcome is listed in Appendix 1.

		Self-rated health				
	Total *n*(%)	Very good *n*(%)	Good *n*(%)	Fair *n*(%)	Poor or very poor *n*(%)	*p*-value
All-cause mortality	6004 (12.7)	2223 (11.5)	2485 (12.7)	997 (15.2)	299 (16.3)	< 0.001
Cancer (total) Stomach cancer Lung cancer Prostate cancer Colon cancer	2238 (4.7) 73 (0.2) 397 (6.6) 194 (0.4) 298 (0.6)	842 (4.4) 29 (0.2) 134 (6.0) 69 (0.4) 110 (0.6)	945 (4.8) 26 (0.1) 183 (7.4) 88 (0.5) 125 (0.6)	356 (5.4) 14 (0.2) 65 (6.5) 30 (0.5) 51 (0.8)	95 (5.2) 4 (0.2) 15 (5.0) 7 (0.4) 12 (0.7)	0.003 0.458 0.191 0.496 0.334
CVD (total) CHD Stroke	2643 (5.6) 1305 (2.8) 100 (1.7)	966 (5.0) 462 (2.4) 35 (1.6)	1096 (5.6) 536 (2.7) 50 (2.0)	441 (6.7) 236 (3.6) 12 (1.2)	140 (7.6) 71 (3.9) 3 (1.0)	< 0.001 < 0.001 0.258
Violent death (total) Suicide Accidents	1014 (2.1) 338 (0.7) 683 (1.4)	387 (2.0) 128 (0.7) 260 (1.4)	406 (2.1) 132 (0.7) 277 (1.4)	169 (2.6) 58 (0.9) 114 (1.7)	52 (2.8) 20 (1.1) 32 (1.7)	0.007 0.058 0.089
Alcohol-related death	803 (1.7)	306 (1.6)	307 (1.6)	158 (2.4)	32 (1.7)	< 0.001

Self-rated health at the time of conscription significantly predicted all-cause mortality during the last 23 years of the 50 years follow-up in a dose response fashion (p for trend = 0.0005) so that conscripts that rated their health as fair or poor/very poor had significantly higher hazard of death than conscript that rated their health as very good (HR 1.27 95%CI:1.18–1.37 and HR 1.25 95%CI:1.11–1.41, respectively, see Table 3). This association was independent of socioeconomic position in 1990 and health status markers at the time of conscription. In conscripts with fair health, the association also remained after further adjustment (HR 1.08 95% CI:1.00–1.17) while the association in conscripts with poor health was rendered non-significant in the full model (HR 0.94 95%CI:0.83–1.07).

**Table 3:** Crude and adjusted hazard ratios (HR) with 95% confidence intervals (CI) for the association between self-reported health and all-cause and cancer mortality (between 1997–2019).

	Crude HR (95%CI)	Model 1 SEP 1990 only Adjusted HR (95%CI)	% Δ	Model 2 Psychological factors Adjusted HR (95%CI)	% Δ	Model 3 Health Status Adjusted HR (95%CI)	% Δ	Model 4 Health Behaviors Adjusted HR (95%CI)	% Δ	Model 5 Total Adjusted HR (95%CI)	% Δ
All-cause mortality Very Good Good Fair Poor or very poor	1.00 1.08 (1.02, 1.14) 1.27 (1.18, 1.37) 1.25 (1.11, 1.41)	1.00 1.07 (1.01, 1.13) 1.34 (1.15, 1.33) 1.19 (1.05, 1.34)	15 13 26	1.00 1.04 (0.98, 1.10) 1.15 (1.06, 1.24) 1.01 (0.89, 1.15)	49 47 94	1.00 1.06 (1.00, 1.12) 1.23 (1.14, 1.33) 1.18 (1.04, 1.32)	23 16 29	1.00 1.02 (0.97, 1.09) 1.14 (1.06, 1.23) 1.06 (0.94, 1.20)	68 47 75	1.00 1.00 (0.94, 1.06) 1.08 (0.99, 1.16) 0.94 (0.83, 1.07)	99 72 100*
Cancer (Total) Very Good Good Fair Poor or very poor	1.00 1.11 (1.01, 1.22) 1.27 (1.12, 1.44) 1.22 (0.99, 1.51)	1.00 1.11 (1.01, 1.21) 1.24 (1.10, 1.41) 1.17 (0.95, 1.45)	3 10 23	1.00 1.07 (0.98, 1.18) 1.14 (1.01, 1.30) 1.00 (0.80, 1.25)	34 48 100	1.00 1.10 (1.01, 1.21) 1.23 (1.09, 1.40) 1.16 (0.95, 1.43)	6 13 28	1.00 1.06 (0.97, 1.16) 1.15 (1.01, 1.30) 1.04 (0.84, 1.29)	45 46 81	1.00 1.03 (0.94, 1.14) 1.07 (0.94, 1.22) 0.92 (0.73, 1.15)	69 75 100*
Stomach cancer Very Good Good Fair Poor or very poor	1.00 0.88 (0.52, 1.50) 1.45 (0.77, 2.75) 1.49 (0.52, 4.29)	1.00 0.88 (0.52, 1.49) 1.44 (0.76, 2.73) 1.52 (0.53, 4.33)	- 2 + 7	1.00 0.87 (0.51, 1.48) 1.37 (0.70, 2.69) 1.42 (0.47, 4.29)	- 17 13	1.00 0.87 (0.51, 1.48) 1.41 (0.75, 2.68) 1.49 (0.52, 4.26)	- 9 0	1.00 0.84 (0.49, 1.42) 1.32 (0.69, 2.52) 1.33 (0.46, 3.83)	- 29 32	1.00 0.82 (0.48, 1.41) 1.26 (0.64, 2.47) 1.26 (0.41, 3.83)	- 43 47
Lung cancer Very Good Good Fair Poor or very poor	1.00 1.35 (1.08, 1.69) 1.44 (1.07, 1.94) 1.21 (0.71, 2.0)	1.00 1.34 (1.07, 1.67) 1.38 (1.02, 1.88) 1.13 (0.66, 1.92)	4 14 38	1.00 1.28 (1.02, 1.61) 1.25 (0.92, 1.72) 1.01 (0.58, 1.76)	19 41 95	1.00 1.33 (1.96, 1.66) 1.36 (1.01, 1.84) 1.09 (0.64, 1.87)	6 17 56	1.00 1.21 (0.97, 1.52) 1.17 (0.87, 1.58) 0.90 (0.53, 1.54)	39 61 100*	1.00 1.18 (0.94, 1.47) 1.10 (0.80, 1.50) 0.83 (0.47, 1.44)	50 78 100*
Prostate cancer Very Good Good Fair Poor or very poor	1.00 1.26 (0.92, 1.73) 1.32 (0.86, 2.02) 1.10 (0.51, 2.40)	1.00 1.25 (0.91, 1.72) 1.29 (0.84, 1.98) 1.08 (0.49, 2.35)	4 9 24	1.00 1.22 (0.88, 1.68) 1.20 (0.77, 1.88) 0.96 (0.43, 2.15)	17 36 100*	1.00 1.26 (0.92, 1.72) 1.31 (0.85, 2.00) 1.09 (0.50, 2.39)	2 4 12	1.00 1.22 (0.89, 1.67) 1.20 (0.78, 1.86) 0.96 (0.44, 2.11)	18 35 100*	1.00 1.20 (0.87, 1.65) 1.17 (0.74, 1.83) 0.91 (0.40, 2.06)	24 46 100*
Colon cancer Very Good Good Fair Poor or very poor	1.00 1.12 (0.87, 1.45) 1.40 (1.00, 1.95) 1.18 (0.65, 2.14)	1.00 1.12 (0.87, 1.45) 1.39 (0.99, 1.93) 1.16 (0.64, 2.11)	0 3 9	1.00 1.13 (0.87, 1.46) 1.38 (0.98, 1.96) 1.14 (0.61, 2.12)	+ 3 4 22	1.00 1.12 (0.87, 1.45) 1.38 (0.99, 1.92) 1.17 (0.64, 2.12)	1 5 8	1.00 1.11 (0.86, 1.43) 1.33 (0.95, 1.87) 1.12 (0.62, 2.05)	14 16 32	1.00 1.11 (0.86, 1.44) 1.34 (0.94, 1.90) 1.12 (0.60, 2.09)	9 16 33

Δ attenuation, representing the proportion in percent of the self-rated health and mortality association explained by the risk factor in question. All values above 100 are marked with “100*” as the attenuation cannot be more than 100%. “+” denotes an increase in HR suggesting an augmentation of the association. “-“ is used when there is no association between self-rated health and mortality in the crude model (HR < 1) as it is not possible to calculate attenuation in this case.

Model 1: adjusted for SEP 1990.

Model 2: Model 1 + adjustments for factors related to psychological factors (low emotional control, psychiatric diagnosis, medication for nervousness).

Model 3: Model 1 + adjustments for factors related to health status (high blood pressure, erythrocyte sedimentation rate (ESR) and high body mass index).

Model 4: Model 1 + adjustments for factors related to health behaviors (risky alcohol use, smoking and cardiovascular fitness).

Model 5: Fully adjusted.

By and large, conscripts with good, fair or poor/very poor self-rated health had a higher hazard of cancer mortality independent of psychological factors and health status markers (Table 3). However, adjustment for health behaviors rendered the association between self-rated health and cancer mortality non-significant. An increased hazard for lung cancer was seen in conscripts with good or fair but not poor self-rated health and was explained by psychological factors and health behaviors. No statistically significant association was seen between self-rated health and colon, stomach or prostate cancer mortality.

Conscripts with good, fair or poor/very poor self-rated health had a higher hazard for CVD and CHD that remained after adjustment for socioeconomic position and health status markers but was explained by the combined adjustment of psychological factors and health behavior, apart from for fair or poor/very poor health that remained associated with a higher hazard of CHD, and the three factors together explained 82–93% of the association with CVD mortality accounting from 16–68% of the association each (Table 4). Self-rated health was not associated with the hazard of death due to stroke.

No statistically significant association was seen between poor self-rated health and increased risk of violent deaths although the general pattern seemed similar to the other causes of death (Tables 4). Conscripts with fair but not poor health had a higher hazard of alcohol related deaths than conscripts with very good health. This association remained after adjustment of socioeconomic position, psychological factors, health status and health behaviors respectively but was rendered non-significant in the full model.

**Table 4:** Crude and adjusted hazard ratios (HR) with 95% confidence intervals (CI) for the association between self-reported health and cardiovascular, violent and alcohol-related deaths (between 1997–2019). For model explanations, see table 3.

	Crude HR (95%CI)	Model 1 SEP 1990 only Adjusted HR (95%CI)	% Δ	Model 2 Psychological factors Adjusted HR (95%CI)	% Δ	Model 3 Health Status Adjusted HR (95%CI)	% Δ	Model 4 Health Behaviors Adjusted HR (95%CI)	% Δ	Model 5 Total Adjusted HR (95%CI)	% Δ
CVD Very Good Good Fair Poor or very poor	1.00 1.12 (1.03, 1.22) 1.38 (1.23, 1.54) 1.57 (1.31, 1.87)	1.00 1.11 (1.02, 1.21) 1.32 (1.18, 1.48) 1.46 (1.22, 1.74)	7 14 19	1.00 1.07 (0.98, 1.16) 1.18 (1.05, 1.33) 1.18 (0.98, 1.43)	46 53 68	1.00 1.10 (1.01, 1.20) 1.29 (1.16, 1.56) 1.43 (1.19, 1.71)	16 22 25	1.00 1.06 (0.97, 1.15) 1.20 (1.07, 1.34) 1.26 (1.05, 1.51)	53 48 55	1.00 1.01 (0.93, 1.11) 1.07 (0.95, 1.20) 1.04 (0.86, 1.26)	90 82 93
CHD Very Good Good Fair Poor or very poor	1.00 1.14 (1.01, 1.30) 1.54 (1.32, 1.80) 1.66 (1.29, 2.13)	1.00 1.14 (1.01, 1.29) 1.48 (1.26, 1.73) 1.53 (1.19, 1.97)	6 12 19	1.99 1.10 (0.97, 1.25) 1.35 (1.15, 1.60) 1.32 (1.01, 1.71)	31 34 52	1.00 1.12 (0.99, 1.27) 1.43 (1.23, 1.69) 1.50 (1.17, 1.93)	16 19 25	1.00 1.08 (0.95, 1.22) 1.33 (1.13, 1.56) 1.31 (1.02, 1.69)	48 39 52	1.00 1.04 (0.92, 1.18) 1.21 (1.03, 1.43) 1.14 (0.87, 1.49)	73 61 79
Stroke Very Good Good Fair Poor or very poor	1.00 1.41 (0.92, 2.18) 1.03 (0.54, 1.99) 0.93 (0.29, 3.01)	1.00 1.40 (0.91, 2.16) 1.01 (0.52, 1.94) 0.88 (0.27, 2.86)	2 82 -	1.00 1.37 (0.89, 2.13) 0.96 (0.49, 1.90) 0.84 (0.25, 2.84)	10 205 -	1.00 1.39 (0.90, 2.15) 0.99 (0.51, 1.91) 0.87 (0.27, 2.84)	4 118 -	1.00 1.36 (0.88, 2.10) 0.95 (0.49, 1.84) 0.83 (0.25, 2.72)	12 241 -	1.00 1.32 (0.85, 2.05) 0.90 (0.45, 1.77) 0.78 (0.23, 2.64)	21 100* -
Violent death Very Good Good Fair Poor or very poor	1.00 1.04 (0.90, 1.19) 1.31 (1.09, 1.57) 1.45 (1.08, 1.93)	1.00 1.03 (0.89, 1.18) 1.25 (1.04, 1.50) 1.32 (0.99, 1.76)	22 19 29	1.00 0.96 (0.83, 1.10) 1.04 (0.86, 1.26) 0.95 (0.70, 1.29)	100* 87 100*	1.00 1.02 (0.89, 1.18) 1.24 (1.03, 1.48) 1.28 (0.96, 1.72)	40 24 36	1.00 0.98 (0.85, 1.13) 1.12 (0.93, 1.35) 1.12 (0.83, 1.50)	100* 61 74	1.00 0.92 (0.80, 1.06) 0.97 (0.80, 1.17) 0.84 (0.62, 1.15)	100* 100* 100*
Suicide Very Good Good Fair Poor or very poor	1.00 1.02 (0.80, 1.30) 1.35 (0.99, 1.85) 1.67 (1.04, 2.68)	1.00 1.01 (0.79, 1.29) 1.29 (0.94, 1.76) 1.51 (0.94, 2.42)	41 19 25	1.00 0.96 (0.75, 1.22) 1.11 (0.80, 1.54) 1.19 (0.72, 1.96)	100* 68 72	1.00 1.00 (0.78, 1.28) 1.28 (0.94, 1.75) 1.48 (0.92, 2.38)	60 20 28	1.00 0.97 (0.76, 1.23) 1.17 (0.85, 1.60) 1.30 (0.81, 2.10)	100* 53 55	1.00 0.92 (0.72, 1.18) 1.05 (0.76, 1.46) 1.09 (0.66, 1.79)	100* 85 87
Accidents Very Good Good Fair Poor or very poor	1.00 1.05 (0.89, 1.25) 1.32 (1.06, 1.64) 1.33 (0.92, 1.92)	1.00 1.04 (0.88, 1.24) 1.26 (1.01, 1.57) 1.22 (0.84, 1.76)	15 18 33	1.00 0.97 (0.81, 1.15) 1.04 (0.82, 1.31) 0.85 (0.58, 1.25)	100* 89 100*	1.00 1.04 (0.88, 1.23) 1.24 (0.99, 1.55) 1.18 (0.81, 1.71)	31 25 44	1.00 0.99 (0.84, 1.18) 1.13 (0.90, 1.41) 1.03 (0.71, 1.50)	100* 60 92	1.00 0.93 (0.78, 1.11) 0.95 (0.76, 1.21) 0.75 (0.51, 1.10)	100* 100* 177
Alcohol-related death Very Good Good Fair Poor or very poor	1.00 0.99 (0.85, 1.16) 1.55 (1.28, 1.88) 1.13 (0.78, 1.62)	1.00 0.98 (0.84, 1.15) 1.45 (1.20, 1.76) 0.97 (0.68, 1.40)	- 18 100*	1.00 0.90 (0.77, 1.06) 1.16 (0.95, 1.42) 0.67 (0.46, 0.98)	- 71 100*	1.00 0.98 (0.84, 1.15) 1.46 (1.20, 1.77) 0.98 (0.68, 1.42)	- 17 100*	1.00 0.90 (0.77, 1.06) 1.22 (1.00, 1.48) 0.75 (0.52, 1.09)	- 61 100*	1.00 0.86 (0.73, 1.01) 1.06 (0.86, 1.30) 0.59 (0.41, 0.87)	- 89 100*

Investigating the last two decades of follow-up separately, in the crude model self-rated health was still a predictor of all-cause mortality and CVD both 30 to 40 years later and 40 to 50 years later, and a predictor of violent death 30 to 40 years later. These associations were rendered non-significant after adjustment by the covariates (Table 5).

**Table 5:** Crude and fully adjusted HRs and 95% CI for the association between poor self-rated health and all-cause and cause-specific mortality, 1997–2008 and 2009–2019, respectively.

	Between 1997-2008^a^		Between 2009-2019^b^	
	Poor or very poor self-rated health Crude HR (95%CI)	Poor or very poor self-rated health Adjusted HR (95%CI)	Poor or very poor self-rated health Crude HR (95%CI)	Poor or very poor self-rated health Adjusted HR (95%CI)
All-cause mortality	1.55 (1.24, 1.94)	0.98 (0.77, 1.23)	1.15 (1.00, 1.33)	0.93 (0.80, 1.09)
Cancer	1.14 (0.73, 1.77)	0.89 (0.56, 1.41)	1.25 (0.98, 1.59)	0.92 (0.72, 1.19)
CVD	1.68 (1.18, 2.39)	1.09 (0.75, 1.58)	1.53 (1.25, 1.88)	1.03 (0.83, 1.28)
Violent	1.60 (1.02, 2.50)	0.81 (0.50, 1.31)	1.35 (0.92, 1.97)	0.87 (0.58, 1.30)
Alcohol-related	0.84 (0.42, 1.65)	0.37 (0.18, 0.75)	1.30 (0.84, 2.00)	0.77 (0.49, 1.22)

Adjusted for SEP 1990, low emotional control, psychiatric diagnosis, medication for nervousness, high blood pressure, erythrocyte sedimentation rate (ESR), high body mass index, risky alcohol use, smoking and cardiovascular fitness.

^a^Between the age of 46/47/48 years and 57/58/59 years.

^b^Between the age of 58/59/60 years and 68/69/70 years.

(Reference group = very good)

## Discussion

We report that a single measure of self-rated health reported in late adolescence significantly predicts all-cause mortality between 46 to 70 years of age. That is, the predictive ability of self-rated health remained 27 years after the subjective health rating in late adolescence and extended an additional 23 years. The association was seen already at good health, and there was a significant trend for a dose–response association, so that the association between self-rated health and mortality became stronger with poorer self-rated health. Our initial hypothesis suggested that the influence of psychological factors on predictive capacity would diminish as the causes of death changed as the men aged. However, in the present study the psychological factors retained this ability, accounting for 94% of the association between self-rated health and all-cause mortality compared to our previous study, where they accounted for 84%. Overall, it seems psychological factors, health status markers and health behaviors may provide additive mechanisms, so that each of these factors contribute with in part unique information that may explain why poor self-rated health is associated with an increased risk of mortality.

The causes of death changed during the follow-up period with violent deaths accounting for the majority of deaths during the first 20 years^10^, and for one-fifth of deaths during the following 30 years. As expected, deaths due to cancer and cardiovascular disease where more common during the last 23 years of the follow-up as compared with the previous 27 years^10^. In the last 23 years of follow up, poor and/or fair self-rated health statistically significantly predicted cancer and cardiovascular disease, alcohol use disorders but not death due to violent causes, the latter likely explained by the few cases in the comparison. The predictive ability of self-rated health decreased with time but the single measure of self-rated health in late adolescence, when the participants had few diagnoses, still predicted mortality with a follow up period of 50 years. We divided the follow-up period into two to see if the predictive ability changed during the last two decades (1997–2008 and 2009–2019) of follow-up. The results were similar between the two time-period, with self-rated health a significant predictor for all-cause mortality and CVD that was rendered non-significant after adjustment of the full model. The only difference between the two time periods was a crude association with violent death that was only significant in the first time period. From these analyses, no support is given for a change in the predictive ability of self-rated health over the follow-up period analyzed in the present study.

As part of the study, we investigated potential explanatory factors for the predictive ability of self-rated health and included adult socioeconomic position, psychological factors, health status markers and health behaviors. Psychological factors and health behaviors at the time of conscription explained a substantial part of the associations between self-rated health and mortality, each attenuated the association with CVD with about 50%, and the effect of the two factors seem to be additive as the major part of the association was attenuated in the full model. Health status markers (blood pressure, BMI and ESR) explained less of the association (around one fifth of the association with CVD) which is in concordance with other studies showing that the predictive ability of self-rated health is to a large extent independent of objective health status markers^2–4^.

The stability of the predictive ability of self-rated health rated in late adolescence on mortality later in life suggest that there is a stability in either the self-rating of health or its underlying factors over time. This could possibly be due to persisting socioeconomic disadvantages^13^. However, adult socioeconomic position explained little of the association between self-rated health and mortality in the present study. Future studies may investigate other measures of socioeconomic disadvantages in addition to registry data on employment status. Previous studies investigating self-rated health over time have concluded that it is a stable measure^3,8,9^. However, there are also studies showing that a worsening in self-rated health is a stronger predictor of mortality than poor self-rated health itself^14^ why targeting poor self-rated health with interventions to improve longevity should not be ruled out. Explanatory factors were not investigated in their study but it is plausible that changes in psychological factors and/or health behaviors explain the association between worsened health status and increased mortality. Poor self-rated health may be relevant to identify at-risk individuals also at a young age and it is plausible that interventions aimed to improve psychological health in young adults may results in an improved self-rated health that in turn result in an improved longevity. For example, treatment for health anxiety has been found to improve self-rated health^15^, but further studies are required to establish if such changes result in a reduction in mortality.

Strength of the study include the well-characterized study population, the minimal selection bias as 97–98% of all men underwent conscription at the time, and the use of national registries for mortality data. The young age of the participating men and the very long follow-up period make the study unique; the follow-up time of 50 years is to the best of our knowledge the longest investigated for the association between poor self-rated health and increased mortality. Limitations include the low number of men with poor self-rated health and certain cancers, limiting the power of analysis of the group with poor or very poor self-rated health and e.g., colon and prostate cancer, which likely explain why there are sometimes inconsistencies in results between the self-rated health groups and especially the poor health group. The overlap of some of the included covariates may lead to overadjustment of the association between self-rated health and morality which may be reflected in the sometimes more than full attenuation of the association after adjustment. For the assessment of attenuation, a simplified method based on the change in HR was applied as the Cox regression is a poor vehicle to formally calculate indirect effects. No women were included in the present study. Self-rated health was the third strongest mortality predictor in women in the UK biobank^1^ and although similar results may be expected in women, this needs to be investigated.

In conclusion, self-rated health reported in late adolescent predicts all cause, cancer and cardiovascular mortality and death due to alcohol abuse between 47 to 70 years of age. Psychological factors and health behaviors explained a large part of the association between self-rated health and future mortality during this very long follow-up.

## Electronic supplementary material

Below is the link to the electronic supplementary material.

Supplementary Material 1

## Data Availability

The data that support the findings of this study are available from Statistics Sweden, but restrictions apply to the availability of these data, which were used under license for the current study, and so are not publicly available. Data are however available from the authors upon reasonable request and with permission of Statistics Sweden.
