# Supplementary material for: Self-rated health in late adolescence as a predictor for mortality between 46 and 70 years of age
Source: Sci Rep. 2024 Oct 15;14:24103. doi: 10.1038/s41598-024-75158-x (PMC11480418; doi:10.1038/s41598-024-75158-x)
Supplement: Supplementary file 1 — Supplementary Material 1 [file 41598_2024_75158_MOESM1_ESM.docx]

**Appendix 1. Diagnostic codes for outcomes**

|  |  |  |
| --- | --- | --- |
|  | **ICD -9**  **(1987-1996)** | **ICD-10**  **(from 1997)** |
| Cancer (total)  Stomach cancer  Lung cancer  Prostate cancer  Colon cancer | 140-239  151  162  185  153, 154 | C00-C97  C16  C33, C34  C61  C18- C20 |
| CVD (total)  CHD  Stroke | 390-459  410-412  433-434 | I00-I99  I20-I25  I63 |
| Violent death (total)  Suicide  Accidents | 900-989  E950-E959, E980-E989  900-949, 960- 979 | V01-Y98  X60-X84, Y10-Y34  V01-X59, X85-Y09, Y40-Y98 |
| Alcohol-related death | 291, 303, 305.0, 357.5, 425.5, 535.3, 571.0-571.3, E860 or E980 and 980 | E24.4, F10, G31.2, G62.1, G72.1, I42.6, K29.2, K70.0, K85.2, K86.0, R78.0, T51.0, Y90, Y91, Z50.2, Z71.4, Z72.1 |
